# VEGF, apelin and HO-1 in diabetic patients with retinopathy: a correlation analysis

**DOI:** 10.1186/s12886-020-01593-9

**Published:** 2020-08-08

**Authors:** Rensiqin Wu, Zhifeng Zhu, Dandan Zhou

**Affiliations:** Department of Endocrinology, The Affiliated Hospital of Inner Mongolia Medical University, No.1st tunnel north road, Hohhot, 010000 Innermongolia China

**Keywords:** Diabetic retinopathy, VEGF, Apelin, HO-1, Risks

## Abstract

**Background:**

It’s necessary to analyze the role of VEGF, apelin, and HO-1 in patients with type 2 diabetes (T2DM), and to evaluate its relevance to diabetic retinopathy (DR).

**Methods:**

T2DM patients who were treated in our hospital from December 1, 2018 to November 30, 2019 were included. T2DM patients were divided into non-DR (NDR) group, non-proliferative DR (NPDR) group, and proliferative DR (PDR) group. and healthy participants were selected as the control group. The value of VEGF, apelin, and HO1 in predicting PDR were analyzed by receiver operating characteristic (ROC) curve, and the relations of VEGF, apelin, HO-1 and clinical factors in PDR patients were analyzed by Pearson correlation analysis.

**Results:**

A total of 295 participants were included. The level of FPG and HbAlc in PDR group were significantly higher than that of other groups (all *p* < 0.05); the level of VEGF and apelin in PDR group were significantly higher than that of other groups (all *p* < 0.05), but the level of HO-1 in PDR group were significantly less than that of other groups(*p* = 0.017); the AUC of VEGF, apelin, HO-1 and combined use was 0.806(95%CI: 0.779–0.861), 0.819(95%CI: 0.765–0.878), 0.808(95%CI: 0.733–0.869) and 0.902(95%CI: 0.822–0.958) respectively, the AUC, sensitivity, specificity of the three combined use was significantly higher than that of single VEGF, apelin, HO-1 use(all *p* < 0.05). The cutoff values of serum VEGF, apelin, and HO-1 levels for predicting PDR were 163.85 pg/ml, 8.27 ng/ml, and 26.06 mmol/L respectively. Serum VEGF, apelin, and HO-1 in patients with PDR was related to the time course of DM, FPG and HbAlc (all *p* < 0.05).

**Conclusions:**

VEGF, apelin and HO-1 are related to the progress of DR, and the combined use of VEGF, apelin and HO-1 is beneficial to the diagnosis and treatment of PDR.

## Background

With the improvement of economic level, the aging related to the increase of average life expectancy, lifestyle has changed significantly changes such as sedentary and unbalanced diets [1]. Many of these factors have caused the number of patients with diabetes mellitus (DM) to increase year by year [2]. It is estimated that by 2025, there will be 300 million people with diabetes worldwide, most of which will be in developing countries [3, 4]. Diabetic retinopathy (DR) is a common and serious complication of diabetes, and its incidence is closely related to the rising prevalence of diabetes [5]. For patients with the duration of diabetes more than 15 years, more than 80% patients will have DR [6]. It’s been reported that the incidence of DR is 39% in patients with type II diabetes who do not need insulin treatment, and the incidence of DR in patients with type II diabetes who require insulin is 70% [7, 8]. Proliferative diabetic retinopathy (PDR) is a middle-to-advanced DR, which is the main cause of severe visual impairment and even blindness [9]. The incidence of PDR increased from 0 to 25% during the course of 3–15 years of diabetes [10, 11]. Therefore, the prevention and treatment of PDR is essential to the prognosis of patients with diabetes.

The main pathological changes of DR are retinal hemorrhage, exudation, microvascular dilatation and neovascularization, but the specific pathogenesis is not yet clear [6]. Recent studies have found that VEGF is involved in retinal exudation, bleeding, and retinal neovascularization, and its level changes have a certain correlation with the occurrence and development of DR [12–14]. Apelin is a new type of adipokine, it’s also a kind of angiotensin receptor-related protein [15]. It’s been reported that Apelin is involved in the proliferation of retinal endothelial cells and the angiogenesis process [16]. Furthermore, previous studies have reported that heme oxygenase 1 (HO-1) abnormally expressed in retinal cells cultured in high glucose and in the retina of diabetes animal models [17, 18]. It’s well-proven that VEGF, apelin, HO-1 are related to many physiological and pathological processes related to immune response such as vascular homeostasis, cell proliferation and apoptosis, and inflammation, but its relationship with DR has not yet been clarified [19, 20]. It’s necessary to further clarify the role of VEGF, apelin, and HO-1 in patients with diabetes. Therefore, we attempted to conduct this study to elucidate the role of VEGF, apelin and HO-1 in diabetes II (T2DM) patients, to provide evidence for the prevention and treatment of DR.

## Methods

### Ethical consideration

Our study has been approved by the ethics committee of the Affiliated Hospital of Inner Mongolia Medical University (20181015), and written consents were obtained from all included patients.

### Participants

T2DM patients who were treated in the department of endocrinology of our hospital from December 1, 2018 to November 30, 2019 were included. The inclusion criteria were as follows: (1) The diagnosis of T2DM met the DM diagnosis guidelines and classification standards [21–23];(2) The patient did not use cytotoxic agents and immunosuppressive drugs, and had no history of taking medication in the past 4 weeks, and had a normal white blood cell count;(3) The patient was informed and agreed to participate in this present study. The exclusion criteria were as follows: (1) Type 1 DM and other types of DM; (2) patients with acute and chronic infections, liver and kidney disease, rheumatic connective tissue disease, cardio-cerebral vascular disease, and malignant tumors; (3) Patients with ocular diseases or fundus lesions that cannot be graded other than DR; (4) Patients who refuse to cooperate and follow up.

All included T2DM patients underwent vision, slit-lamp microscope, indirect ophthalmoscope, and fundus fluorescein angiography (FFA) examination. With reference to the clinical diagnostic criteria of DR, the included T2DM patients were divided into non-DR (NDR) group, non-proliferative DR (NPDR) group, and proliferative DR (PDR) group based on the test results.

Additionally, healthy people who underwent physical examination in our hospital during the same period were selected as the control group. For this group, we exclude those with systemic and ocular diseases, and those with a body mass index (BMI) ≥ 25 kg/m^2^.

### Laboratory analysis

All participants have been taken 5 ml of cubital vein blood on an status of empty stomach. After centrifugation, the serum was collected and stored in a − 70 °C refrigerator. ELISA was used to determine the level of serum VEGF, apelin, HO-1. The detection kit was purchased from Hensheng biomedicine company (Shanghai, China). all the operation was strictly performed in accordance with the manual instructions of related kits.

### Data collections

We collected and recorded the participants’ age, gender, duration of DM, blood pressure, height, weight, BMI, fasting blood glucose (FPG), glycated hemoglobin (HbAlc), total cholesterol (TC), triglycerides (TG), Total cholesterol (TC), high density lipoprotein cholesterol (HDL-c), low density lipoprotein cholesterol (LDL-c) and other laboratory indicators.

### Statistical analysis

Statistical analysis was performed using SPSS 23.0 software. Measurement data are expressed as mean ± standard deviation (M ± SD). One-way analysis of variance was used for comparison between multiple groups, and SNK test was used for pairwise comparison within groups; Group t test was used for comparison between two groups. Chi-square test was used for comparison of count data. According to the receiver operating characteristic (ROC) curve, the thresholds for predicting the occurrence of PDR by VEGF, apelin, and HO-1 were determined. The determination of the critical values ​​was based on the maximum Jordanian index (sensitivity + specificity-1). The area under the ROC curve (AUC) was used to compare the predictive value of related factors to the occurrence of PDR, and to determine its sensitivity and specificity, positive and negative predictive values. In this present study, AUC less than 0.8 was considered as low predicted value, a AUC within 0.8 to 0.9 was considered as medium predicted value, and AUC > 0.9 was considered as high predicted value. The correlation analyses on the serum VEGF, apelin, HO-1 with clinical indicators were conducted with Pearson correlation analysis methods. In this present study, *P* < 0.05 was considered statistically significant.

## Results

### The characteristics of included participants

A total of 295 participants were included in this preset study, with 80 controls, 75 NDR patients, 72 NPDR patients and 68 PDR patients respectively (Table 1). There was significant difference in the time course of DM(*p* = 0.021) among DM groups, no significant differences were found in the gender, age, BMI and cases of hypertension among groups (all *p* > 0.05).

**Table 1:** The characteristics of included participants

Groups	Cases	Male/female	Age(y)	Time course of DM(y)	BMI (kg/m^2^)	Hypertension (%)
Control	80	45/35	57.9 ± 3.95	–	22.5 ± 3.04	22 (27.5%)
NDR	75	41/34	57.4 ± 2.57	2.3 ± 0.82	22.2 ± 2.99	26 (34.67%)
NPDR	72	38/34	56.9 ± 3.88	4.3 ± 1.05	22.5 ± 3.13	26 (36.1%)
PDR	68	37/29	57.4 ± 3.36	7.2 ± 2.44	22.4 ± 3.07	24 (35.29%)
χ^2^/F		0.883	10.095	1.371	0.538	1.366
p		0.114	0.098	0.021	0.185	0.052

### The HDL-c, LDL-c, TG, TC, FPG, HbAlc level

As Table 2 presented, there were significant differences in the FPG, HbAlc level among groups, and the level of FPG and HbAlc in PDR group were significantly higher than that of other groups (all *p* < 0.05), and no significant differences were found in the HDL-c, LDL-c, TG, TC level among groups (all *p* > 0.05).

**Table 2:** The HDL-c, LDL-c, TG, TC, FPG, HbAlc level among groups

Groups	Cases	HDL-c (mmol/L)	HDL-c (mmol/L)	TG (mmol/L)	TC (mmol/L)	FPG (mmol/L)	HbAlc(%)
Control	80	2.37 ± 0.22	2.57 ± 0.32	1.58 ± 0.21	4.31 ± 0.61	5.29 ± 0.97	5.11 ± 0.68
NDR	75	2.36 ± 0.30	2.49 ± 0.35	1.59 ± 0.28	4.66 ± 0.68	6.02 ± 1.33^*^	8.95 ± 1.80^*^
NPDR	72	2.29 ± 0.36	2.50 ± 0.41	1.72 ± 0.31	5.03 ± 0.84	9.15 ± 2.16^*#^	9.84 ± 2.15^*#^
PDR	68	2.30 ± 0.38	2.44 ± 0.35	1.74 ± 0.28	5.79 ± 0.78	11.80 ± 3.19^*#&^	10.17 ± 3.32^*#&^
F		1.395	0.988	0.852	1.096	10.468	9.395
p		0.275	0.107	0.097	0.145	0.037	0.011

Notes: ^*^p < 0.05 compared with Control; ^#^*p* < 0.05 compared with NDR; ^&^*p* < 0.05 compared with Control

### The VEGF, apelin and HO-1 level

As Table 3 presented, there were significant differences in the VEGF, apelin and HO-1 level among groups, and the level of VEGF and apelin in PDR group were significantly higher than that of other groups (all *p* < 0.05), but the level of HO-1 in PDR group were significantly less than that of other groups(*p* = 0.017).

**Table 3:** The VEGF, apelin and HO-1 level among groups

Groups	Cases	VEGF (pg/ml)	Apelin (ng/ml)	HO-1(mmol/L)
Control	80	57.03 ± 9.49	2.09 ± 0.82	50.38 ± 9.15
NDR	75	89.15 ± 10.44^*^	4.22 ± 1.04^*^	41.06 ± 8.52^*^
NPDR	72	153.25 ± 20.58^*#^	6.58 ± 1.86^*#^	33.79 ± 6.15^*#^
PDR	68	186.50 ± 30.16^*#^	9.16 ± 2.33^*#^	25.19 ± 5.27^*#^
F		28.573	20.805	16.187
p		0.001	0.003	0.017

Notes: ^*^p < 0.05 compared with Control; ^#^*p* < 0.05 compared with NDR; ^&^*p* < 0.05 compared with Control

### The diagnosis value of VEGF, apelin, HO-1 for PDR

**Fig. 1:**
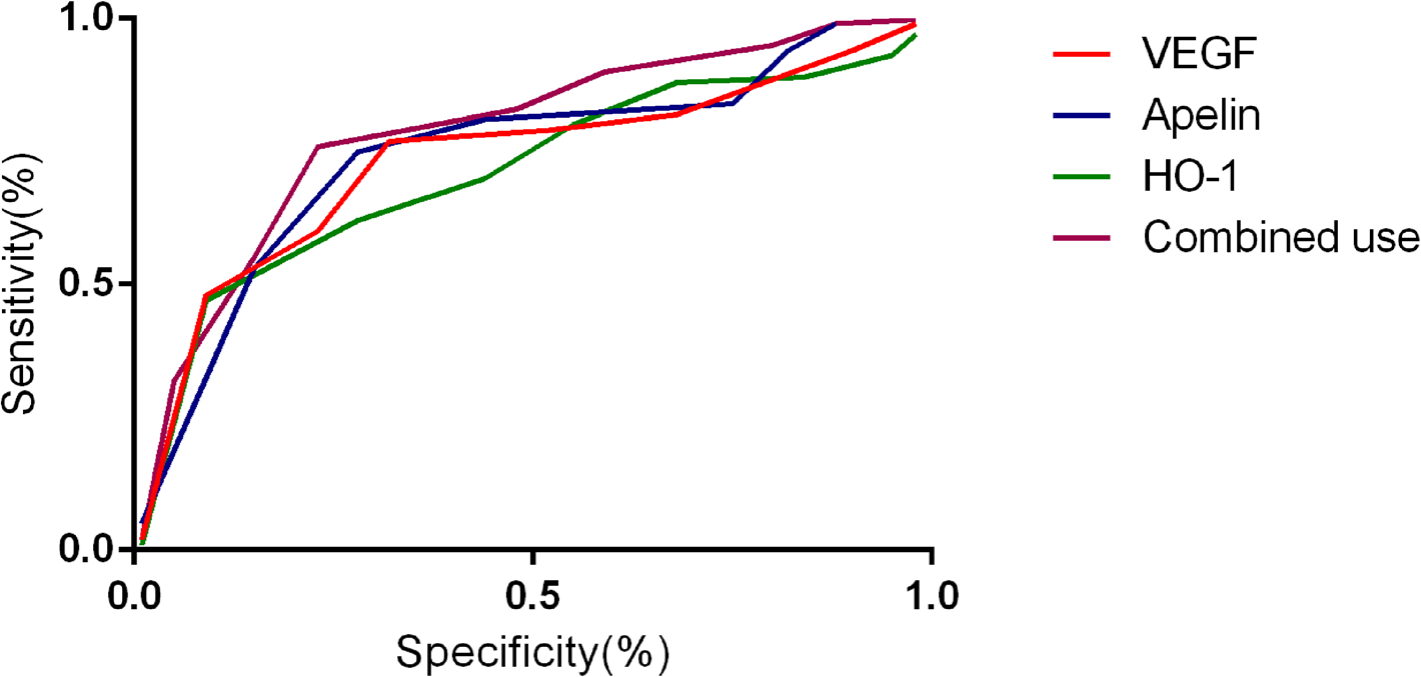
The ROC curve of VEGF, apelin, HO-1 for predicating PDR

As Fig. 1 and Table 4 showed, the AUC of VEGF, apelin, HO-1 and combined use was 0.806(95%CI: 0.779–0.861), 0.819(95%CI: 0.765–0.878), 0.808(95%CI: 0.733–0.869) and 0.902(95%CI: 0.822–0.958) respectively, the AUC, sensitivity, specificity of the three combined use was significantly higher than that of single VEGF, apelin, HO-1 use(all *p* < 0.05). The cutoff values of serum VEGF, apelin, and HO-1 levels for predicting PDR were 163.85 pg/ml, 8.27 ng/ml, and 26.06 mmol/L respectively.

**Table 4:** The analysis on the diagnosis value of VEGF, apelin, HO-1 for PDR

Items	Cutoff	AUC	95%CI	p	Sensitivity(%)	Specificity(%)	Positive predictive value(%)	Negative predictive value(%)
VEGF	163.85	0.806	0.779–0.861	0.021	76.43	85.19	88.42	71.90
Apelin	8.27	0.819	0.765–0.878	0.016	84.08	82.95	85.68	80.62
HO-1	26.06	0.798	0.733–0.829	0.009	73.99	81.42	78.15	78.07
Combined use	–	0.885	0.812–0.918	0.007	90.35	83.18	86.69	85.23

### The risk factors related to PDR

As Table 5 presented, Pearson correlation analysis showed that serum VEGF, apelin, and HO-1 in patients with PDR was related to the time course of DM, FPG and HbAlc (all *p* < 0.05), and the serum VEGF, apelin, and HO-1 in patients with PDR was not related to the age, gender, BMI, hypertension, HDL-c, LDL-c, TG and TC (all *p* > 0.05). Serum VEGF, apelin and HO-1 were significantly correlated with each other (*r* = 0.787, − 6.165, − 5.090 respectively, all *p* < 0.05).

**Table 5:** Correlation analysis on the risk factors related to PDR

Variables	VEGF		Apelin		HO-1	
	r	p	r	p	r	p
Age	0.215	0.180	0.257	0.097	−0.148	0.103
Gender	0.115	0.097	0.150	0.104	−0.121	0.079
Time course of DM	0.389	0.042	0.275	0.033	0.206	0.044
BMI	0.167	0.079	0.201	0.059	0.085	0.102
Hypertension	0.294	0.157	0.175	0.202	0.213	0.119
HDL-c	−0.168	0.096	−0.074	0.138	0.128	0.085
LDL-c	0.156	0.104	0.183	0.099	−0.226	0.142
TG	0.115	0.095	0.245	0.206	−0.117	0.195
TC	0.099	0.183	0.105	0.126	−0.136	0.092
FPG	0.468	0.018	0.381	0.009	−0.403	0.017
HbAlc	0.395	0.037	0.214	0.015	−0.378	0.035
VEGF	–	–	0.787	0.011	−6.165	0.017
Apelin	0.787	0.011	–	–	−5.090	0.009

## Discussion

Retinal microvessels are exposed to high glucose and hypoxic environments for a long time in DM patients, and retinal sensory nerve function damage, and the changes in microvascular structure and function will occur, leading to the onset and development of DR [24]. With the progress of the disease, the retinal microvessels gradually developed from early aneurysm-like changes to the formation of new blood vessels. The walls of newly generated microvessels are fragile and lack the oxygen supply. It is easy to rupture under the action of pulling, causing severe consequences such as vitreous hemorrhage and retinal detachment, and it can eventually lead to blindness without effective clinical intervention [14, 25]. Therefore, the early diagnosis and treatment of PDR are very important to the prognosis of DM patients. The results of this present study have revealed that VEGF, apelin, and HO-1 are associated with the development of PDR, and they can be adopted for the diagnosis of PDR with consideration to their good predicating value.

VEGF is a kind of dimeric glycoprotein with heparin-binding activity, and its main role is to promote the proliferation of endothelial cells and induce the formation of new blood vessels [26, 27]. The blood supply to the retina plays an important role in maintaining and protecting the functions of retinal ganglion cells. VEGF can specifically bind to vascular endothelial cell receptors, increase the permeability of blood vessels, aggravate ischemia and hypoxia in local retinal tissues [28]. Previous study has pointed out that there is no correlation between the expression levels of VEGF in blood and vitreous fluid in patients with PDR, and he believes that almost all VEGF in vitreous fluid is generated by the surrounding tissues in the eye [29]. But the study by Baharivand et al. has shown a positive correlation between VGEF levels in blood and vitreous fluid (r = 0.45) [30]. The results of our study have further indicated that VGEF is associated with the development of PDR.

As a fat cytokine, Apelin plays an important role in various pathophysiological processes such as inflammatory response, immune response, cell growth and development, and apoptosis [16, 31]. Recent studies have found that apelin can stimulate retinal endothelial cell proliferation and retinal neovascularization [32–34]. And previous studies have found that apelin receptor is highly expressed in the formation of embryonic blood vessels and the formation of retinal blood vessels in mice [35, 36]. The link between the high expression of this receptor and angiogenesis is closely related to the mitogenic properties of apelin on endothelial cells and the activation of new blood vessels [37]. In the experiments using retinal vascular endothelial cell line RF/6A in vitro, Kasai et al. have found that apelin can significantly promote the proliferation, migration and formation of vascular lumen of RF/6A cells [38]. The process of neovascularization depends on the proliferation and migration of vascular endothelial cells and the formation of capillary-like lumens [39], it is considered that apelin is a new angiogenic factor that has a mitogenic effect on endothelial cells and promotes angiogenesis.

HO-1 is a rate-limiting enzyme with anti-oxidant, anti-inflammatory, anti-apoptotic effects [40]. When cells are stimulated by oxidative stress, oxidized low-density lipoprotein, cytokines and growth factors, and endotoxin, the activity of HO-1 can increase up to 100 times [41]. HO-1 is abundant in the retina, and is found in retinal pigment epithelial cells, microglia and neurons [42]. HO-1 decomposes free heme to bilirubin, ferritin and other antioxidant products, which together form an important endogenous protection system of the body [41]. Through multiple mechanisms such as anti-oxidation, anti-inflammatory damage, regulation of apoptosis and anti-proliferation, HO-1 helps the body’s tissues and organs return to normal homeostasis after various pathological mechanisms are triggered. Previous studies have shown that HO-1 has certain effects on the development of DR [43, 44], especially on dysfunction and death of retinal endothelial cells, which is consistent with our findings.

## Conclusion

The results of this study have indicated that there may be a close relationship between VEGF, apelin, HO-1 and abnormal glucose metabolism, and they may participate in the process of PDR. Previous studies have suggested that serum apelin levels are significantly increased in patients with DR, and it are related to patients with DM, which can be used as a reliable indicator for assessing the progress of DR [45, 46]. It can be seen that the serum levels of VEGF and apelin are high in DR patients, while the expression level of HO-1 is lower. At the same time, the combined detection of VEGF, apelin and HO-1 is helpful to predict the development of PDR. However, limited by sample size, we cannot perform subgroup analysis based on different DR staging, more studies are needed to further explore the role of VEGF、apelin and HO-1 and potential mechanisms in DM.

## Data Availability

All data generated or analyzed during this study are included in this published article.

## Abbreviations

DM: Diabetes mellitus
DR: Diabetic retinopathy
PDR: Proliferative diabetic retinopathy
NDR: Non-diabetic retinopathy
NPDR: Non-proliferative diabetic retinopathy
ROC: Receiver operating characteristic
